# Supplementary material for: Are carbon emissions trading and green financial instruments synergistic? -Comprehensive quantitative research based on content analysis
Source: PLoS One. 2024 Mar 7;19(3):e0298601. doi: 10.1371/journal.pone.0298601 (PMC10919601; doi:10.1371/journal.pone.0298601)
Supplement: S1 Data — (ZIP) [file pone.0298601.s001.zip › Data/地级市Z_F工作报告.zip!/╕ú╜¿/╚¬╓▌╩╨/└┤╫╘╣1⁄2╓┌║┼í╛┬φ┐╦╩2╛▌═°í┐_2015.docx]

各位代表：

　　现在，我代表泉州市人民政府，向大会报告政府工作，请予审议，并请市政协各位委员和其他列席的同志提出意见。

　　一、2014年工作回顾

　　过去的一年，在省委、省政府和市委的坚强领导下，全市上下深入学习贯彻党的十八大、十八届三中、四中全会和习近平总书记系列重要讲话、来闽考察重要讲话精神，扎实开展党的群众路线教育实践活动，深入实施市委“三大战略”，出台并落实科学发展跨越发展行动方案，改革开放和现代化建设迈出新的步伐。

　　——我们把保稳、求进、攻坚作为工作主线，面对复杂严峻的形势，见事早、出手快，有力应对、有效调度，全市实现生产总值5733.36亿元、增长10.1%，经济总量连续16年位居全省首位，跨越发展的基础更加夯实。

　　——我们把改革、开放、创新作为强大动力，“海丝”先行区建设列入国家“一带一路”战略，国家级金融改革得到深化和拓展，民营经济综合改革提升为全国性试点，“企业全方位创新、政府全方位服务”全面展开，跨越发展的活力进一步激发。

　　——我们把建设“影响东亚、面向世界的多元文化都市”作为重要任务，圆满完成东亚文化之都系列活动，成功举办“海丝”国际艺术节，出色完成文化外交责任，“文都”、“海丝”唤起了海内外泉州人最广泛的文化共鸣和倾情参与，闽南文化和泉州文化的世界性、影响力进一步彰显，跨越发展的软实力大幅提升。

　　——我们把大招商、招大商、招好商作为重中之重，启动实施“54321”计划，突出抓好140个、总投资近7000亿元的战略性项目，中化乙烯、富德新型能源化工、莲花汽车等重特大项目成功引进，跨越发展的后劲持续增强。

　　——我们把保障民生、改善民生作为出发点和落脚点，全市财政民生支出354.02亿元，占公共财政预算支出74%。城东、东海、江南三大学园先后启用，中医联合医院、第三医院、医大附属二院新院、儿童医院新院区相继开诊。为民办实事项目全面完成。荣膺全国文明城市称号。跨越发展的成果更多惠及广大人民。

　　这一年，困难比预料的多，收获比预想的大。全市上下齐心协力，奋力拼搏，付出了艰辛的努力，取得了可喜的成绩。

　　（一）突出多措并举，经济增长平稳健康

　　坚持“三个一”协调调度机制，加强研判预警，务实政企互动。出台拓展民综改革、稳定工业增长、扶持小微企业等惠企措施，建立政策传递、对接、兑现“一条龙”服务机制，落实帮扶企业资金45.9亿元；争取上级各类补助106亿元。建立企业资金应急处置联席会议制度，协调处置33家企业的资金链问题；设立企业应急周转专项资金6.58亿元，累计为企业提供“过桥”资金75.07亿元；全市新增信贷资金投放1160亿元。加强项目调度，市政府按月召开协调调度会，320个在建重点项目完成投资930亿元。狠抓市场开拓，深化企业“手拉手”行动，推动对接产能650亿元；持续开展“电子商务在行动”，电商交易总额1420亿元、增长33.7%；举办泉州商品博览会和品牌泉州境外行系列活动。

　　（二）突出创新引领，产业升级步伐加快

　　统筹用好创新资源。整合市、县各项创新资金13亿元，集中扶持400家重点企业。抓好全国“数控一代”和“中国制造2025”试点，启动百个示范项目、百家示范企业。扎实推进中科院泉州装备所建设，引进设立中国工程院国家数控泉州分中心、华中科大泉州智能制造研究院等平台。新增高新技术企业59家和中国驰名商标31件，制修订各级标准87项、授权专利11456件。实现全国科技进步先进市“九连冠”。开展“人才服务年”活动，为重点项目定制人才政策，创新非公企业专业技术人才评选工作，新增院士专家工作站4家、省级以上引智项目6个。实施产业龙头促进计划。推动中化炼油、恒安智能化、晶安光电（二期）等90个大项目建成投产，完成技改投资637.66亿元，新增百亿企业1家、亿元企业171家。规模以上工业产值突破1万亿元。超千亿产业增加到5个。轻重工业比重进一步优化，重工业比重提高1.6个百分点。完成海洋生产总值1605亿元、增长13.5%。突出上门招商、产业链招商，务实组织和参加多场招商活动，共签约项目389个，总投资4200多亿元。大力发展现代服务业。出台落实扶持商业模式创新、工业设计、快递业发展等政策。“第三产业发展年”433个项目完成投资728.6亿元，国际轻纺城（一期）、南安公路港、EC产业园数据中心等建成，海峡石化交易中心开业。获批筹建“中国海洋产权交易中心”、“中国茶业交易中心”。快递业务量达2.59亿件、增长66%。全市接待游客4428.26万人次，旅游总收入528.65亿元，分别增长18.7%和18.3%。

　　（三）突出先行先试，改革开放扎实推进

　　金融改革持续深化。出台落实金融业发展五年规划等政策文件，获准开展泉台跨境人民币贷款等3项试点。完成金改项目46个。入选全国小微企业信用体系建设试验区，率先建成中小微企业信用信息交换共享平台。小微企业信贷覆盖率达31.9%，比上年末提高6.9个百分点，比金改前提高16.9个百分点。消费金融公司、海峡金融资产交易中心开业，企业集团财务公司获批筹建，金融租赁公司申报筹建。新增上市企业9家、新三板挂牌企业4家，海峡股权泉州交易中心挂牌企业307家。出台民间融资管理规定，推动民间融资登记备案。严厉打击逃废债和非法金融活动，有效防控金融风险。银行业金融机构不良贷款率1.05%，全省最低。民综改革不断拓展。制订民综改革三年实施计划，突出抓好10个方面182项改革，推进各县（市、区）主题改革。启动工商登记制度改革，全年新登记市场主体9.6万家、增长48.2%。探索建立负面清单管理模式，完成民间投资2084.55亿元、增长24.7%。出台批而未用和闲置土地的处理措施，盘活存量土地1793公顷。外向型经济加快发展。完成外贸出口181.81亿美元、增长10.4%，位居“中国外贸百强市”前列。关检“三个一”等便利通关模式全面推行。完成港口吞吐量1.12亿吨、集装箱188万标箱，新增外贸集装箱航线5条。泉州港石井作业区口岸获准对外开放，中化斗尾30万吨级码头建成。泉州晋江国际机场旅客吞吐量达278万人次，新增国际航线2条。出台加快台商投资区开发建设的意见。多方位拓展和深化与台港澳侨的交流合作。

　　（四）突出文化弘扬，名城效应日益凸显

“文都”建设全面铺开。坚持“东亚意识、文化交融、全民参与、全域联动”，组织中韩日交流活动37场、重大文化活动近300场、群众文化活动2万多场。启动实施古城文化复兴计划，建成文都“大麦仓”、泉州木偶剧院，筹备建设市公共文化中心。抓好闽南文化生态保护区20个重点区域整体性保护，新增国家级“非遗”项目3个、中国传统村落4个。积极创建国家公共文化服务体系示范区，扎实推进数字图书馆、博物馆等文化惠民项目，加强档案、社科、方志等工作。文化产业增加值293亿元、增长15%，星期yi服饰创意博览园、洪山文创园等项目建成，永春获评中国香都。“海丝”战略率先启动。主动对接国家“一带一路”战略，编制“海丝”先行区总体方案、战略规划和行动计划，策划实施“海丝”项目180个。成功举办“海丝”国际品牌博览会、首届中阿城市论坛等，牵头发起建立21世纪海上丝绸之路城市联盟和商务合作机制倡议。携手国内“海丝”城市，签署联合申遗“泉州共识”。积极拓展与“海丝”沿线国家、地区的合作，实现贸易总额157.6亿美元。

　　（五）突出城乡统筹，区域发展更加协调

　　城市建管同步加强。深化“环湾规划建设年”活动，427个项目完成投资477亿元，环湾建成区面积197.6平方公里。加快完善环湾区域城乡一体化规划，开展规划督察员试点。实施城乡基础设施提升工程，高速公路通车里程超过500公里，沈海高速复线泉州段、机场连接线建成通车，泉州湾跨海大桥主体工程基本完成，厦沙高速泉州段开工建设，泉州动车站开通始发列车。中心市区新增和优化公交线路12条，新增车位1万多个。实施宜居环境建设行动，开展晋江洛阳江两岸景观整治规划编制，江滨北路绿道慢线全线贯通。完成市政提升“五千工程”787.5公里，建成城市森林生态景观示范片9166亩。综合治理 “两违”，拆除面积422.3万平方米。环湾建成区实现“数字城管”全覆盖。持续深化文明创建活动，扎实推进城市公共环境、公共秩序整治，市民文明素质、城市文明程度进一步提升。

　　新型城镇化有序推开。晋江新型城镇化工作获中央肯定并成为国家级试点，石狮全域城市化、德化统筹城乡、泉港和南安沿海“三镇”产城联动等试点取得新进展。16个镇列入全国重点镇，5个中心镇获批省级“小城市”试点，省市试点镇建设完成投资390.6亿元。启动石结构房屋五年改造计划，完成拆迁1807万平方米、基本建成1103万平方米。新办理流动人口居住证117.9万张，农业转移人口市民化有序推进，全市城镇化率达62.9%。

　　“三农”基础得到夯实。完成农林牧渔业总产值308.88亿元，稳定粮食播种面积215万亩；建成设施农业重点项目80个；远洋渔船总吨位和捕捞产量跃居全省第二。115个水利项目完成投资45.1亿元，白濑水利枢纽项目被列为国家战略性重大水利工程，“七库连通”山美至惠女水库段全面动工，晋江防洪工程、桃溪流域治理等项目部分建成。解决农村23万人饮水安全问题。加强气象服务能力建设。实施公路安保工程953公里，提级改造农村公路137公里，建设升级农村配电网828公里。250个市级“美丽乡村”示范项目完成投资6.35亿元，永春获批全国农村综合改革“美丽乡村”标准化建设试点。大力推进扶贫开发，集中帮扶30个重点乡镇、300个重点村，实现年度脱贫5万人的目标。完成省市造福工程危房改造3434户。加强老区工作，安南永德列入赣闽粤原中央苏区振兴发展规划。

　　（六）突出标本兼治，环境保护成效明显

　　通过国家级生态市技术评估、园林城市复查，获评省级森林城市。实施节能项目100个、减排治理项目1281个，完成节能减排任务。泉港石化园区列入国家循环化改造示范试点。深入开展重点流域、跨境流域污染整治和小流域“赛水质”活动，新建污水处理厂6座、污水管网391公里，新增日污水处理能力12万吨，省级以上工业园区全面实现污水集中处理。大力推进山美水库生态保护国家试点项目建设，加强惠女水库周边畜禽养殖污染整治，全市禁养区的畜禽养殖场基本关闭或拆除。做好PM2.5监测发布，实施大气污染治理项目20个，市区空气优良率达95.3%。完成植树造林11.59万亩、水土流失治理28.48万亩、矿山恢复治理698.6万平方米。

　　（七）突出民生优先，保障水平稳步提高

　　社保继续提标扩面。新增城镇就业15.6万人，农村劳动力转移就业6.1万人。城镇职工“五险”参保人数累计增加38.2万人次。城镇居民医保和新农合参保率分别达95%和99.96%。社会救助体系进一步完善，救助困难群众7.19万人，惠及低保对象13.6万人。城乡居民基本养老保险参保率达99.2%。新增养老床位9000张，建设农村老年体育活动中心20个，建成江南老年颐乐园和市福乐家园（一期）。加强老龄、老干部和关心下一代工作。开工建设保障性安居工程3.54万套，新增配租配售1.13万套；新归集住房公积金49.2亿元，发放贷款36.5亿元；中心市区安置房办证完成产权登记46.7万平方米。全市慈善机构到位善款近9亿元，累计接受侨捐超百亿元。教育致力统筹优化。出台落实统筹优化中心市区教育资源、提升特殊教育等政策，完成义务教育学校标准化建设任务。新扩容城镇中小学学位2.2万个，新增公办幼儿园学位1万个。建成青少年社会教育活动中心20座。华侨大学、泉州师院等高校的办学水平进一步提高，泉州信息学院升格本科。全民健身运动深入开展。我市运动员参加省运会等重大赛事取得好成绩。卫生着力改革提升。实现市县两级公立医院综合改革全覆盖，石狮、晋江成为全国试点，药品销售零差率全面推行。新增医疗机构床位2500张。加强基层卫生服务和疾病预防工作。有效防控人感染H7N9流感。落实人口计生 “单独两孩”政策，生育水平保持稳定。妇女儿童发展纲要深入实施。“平安泉州”建设不断深化。完善维稳应急处突机制，构筑治安立体防控“七张网”，保持对违法犯罪严打高压态势，健全社区矫正制度，社会治安持续向好。荣获全国“六五”普法中期先进城市。完善“大调解”工作体系，严格落实信访“路线图”，有效化解信访积案。认真执行市人大进一步加强食品安全监督管理议案的决议，启动食品放心工程三年行动，深化食品药品监管体制改革。率先开展安全生产标准化提升工程，有效排查治理安全生产隐患，加强道路交通安全综合整治，事故四项指数持续下降。切实维护宗教和谐和民族团结。不断完善防震减灾工作体系。深化军民融合发展，海军“泉州舰”命名入列，国防动员、民兵预备役、海防、人防、支前等工作取得新成效。

　　（八）突出作风整肃，政务效能不断提升

　　坚决贯彻“八项规定”，大力纠正“四风”问题，在市委的统一领导下，党的群众路线教育实践活动取得阶段性成果。以市政府名义召开的会议减少12%、下发的文件减少18.2%。机关后勤管理更加科学规范，全市政府系统“三公”经费支出下降36.6%。自觉接受市人大及其常委会监督，依法执行市人大及其常委会决定决议，主动配合市人大常委会开展执法检查及专题询问、人大代表视察及专项调研，办理人大代表建议293件，满意和基本满意率相加达100%。支持政协履行职能，主动接受市政协民主监督，开展重大事项事前协商，办理政协提案519件，满意和基本满意率相加达100%。基本完成新一轮市、县政府机构改革。稳步推进行政权力清理。深化行政审批制度改革，向社会公布市级行政审批事项；市县乡三级行政服务中心全面完成政务服务标准化建设。顺利完成第三次全国经济普查。廉政建设、行政监察、审计监督、机关效能、绩效考评等工作进一步得到加强。

　　各位代表，成绩的取得来之不易，这是在省委、省政府正确领导下，市委统揽全局、科学决策的结果，是全市人民共同努力、不懈奋斗的结果。在此，我谨代表市人民政府，向全市广大干部群众，人大代表、政协委员，各民主党派、工商联、无党派人士、各人民团体、离退休老同志，向驻泉省部属单位、驻泉部队、武警官兵，企业界人士和来泉创业务工人员，向广大港澳同胞、台湾同胞、海外侨胞、海内外朋友们，表示衷心的感谢和崇高的敬意！

　　我们也清醒认识到，我市发展中还存在不少困难和问题。主要有：政府推进经济社会发展的部分政策举措还不完全适应新形势的新要求；地区生产总值、公共财政总收入、公共财政预算收入等部分指标增速与年度预期还有一定差距；产业转型升级不快，高新技术产业规模偏小，新兴产业和现代服务业发展偏慢；中小微企业经营困难，融资、担保、库存、创新不足等问题仍较突出；重大项目还不多，“落地难”问题亟需破解；统筹城乡发展的层次和水平还需提升，新型城镇化步伐有待加快、质量有待提高，中心城市辐射带动能力不强，农业农村基础还比较薄弱；节能减排和生态建设有待加强，食品药品监管、安全生产、社会稳定等方面还存在不少隐患；教育、卫生、养老等民生欠账仍然较多，人均保障水平不高；政府的少部分公务人员缺乏担当勇气和落实意识，不作为、慢作为等“四风”问题在一些部门、一些干部身上仍不同程度存在，等等。我们一定要高度重视这些问题，采取更加有力措施认真加以解决。

　　二、扎实做好2015年工作

　　今年是全面深化改革、全面推进依法治市、全面完成“十二五”规划的重要一年。做好今年工作，要深刻理解和正确把握中央对我国经济发展进入新常态和对国际形势的科学研判，深刻理解和认真落实省委、省政府建设新福建的一系列部署要求，把握难得的历史新机遇，充分发挥泉州独特优势和基础潜力，砥砺奋进，乘势而上，力争在新常态下有新的作为，在更高起点上实现新的跨越。

　　政府工作的总体要求是：深入贯彻党的十八大和十八届三中、四中全会精神，认真落实习近平总书记系列重要讲话和来闽考察重要讲话精神，紧紧围绕“四个全面”战略布局，按照市委“三大战略”部署要求，全力实施科学发展跨越发展行动方案，加快建设“海丝”先行区，进一步拓展提升“晋江经验”，大力营造有利于大众创业、万众创新的政策环境和社会氛围，推动产业发展、城乡建设、改革开放、文化融合、生态文明、民生改善等方面在全省跨越发展中打头阵、走前列，打造机制活、产业优、百姓富、生态美的现代化泉州。

　　经济社会发展的主要预期目标是：全市生产总值增长10.5%左右，农业总产值持平，工业增加值增长12%；一般公共预算总收入增长10.5%左右，一般公共预算收入增长9.5%左右；全社会固定资产投资增长20%左右；外贸出口增长8%，实际利用外资增长8%；城镇登记失业率控制在2%以内；社会消费品零售总额增长13%，居民消费价格涨幅控制在3%左右；人口自然增长率控制在9.5‰以内；城镇居民人均可支配收入增长8.5%，农民人均可支配收入增长9.5%；完成省下达的节能减排任务。顺利实现“十二五”规划目标。重点抓好以下七个方面工作：

　　（一）大力推进创新转型，进一步培育新的经济增长点

　　挖掘增长动力。梳理整合惠企政策和资金，调整扶持方向和重点，进一步出台更管用的措施。发挥投资关键作用。扎实推进跨越发展“54321”计划，实施年度重点项目475个、总投资9743亿元，完成年度投资980亿元以上，其中超50亿元项目35个、超百亿元项目14个。进一步拓展项目源，着力对接一批大型央企、知名民企和重大外资项目。全力抓好富德新型能源化工、莲花汽车等重特大项目。强化“一月一协调、一季一督查”，推进和谐征迁，积极通过政银企对接、产业基金、PPP、保险资金、债券融资等方式，多方筹措建设资金。发挥消费基础作用。实施养老健康家政、信息、旅游、住房、绿色、教育文化体育等6个领域消费工程，积极发展个性化、多样化消费。加快“智慧城市”建设，扩大信息消费。完善城乡流通网络，营造诚信安全的消费环境。发挥出口拉动作用。落实促进外贸发展政策，办好各类经贸活动。推动重点出口基地转型升级，扩大自主知识产权、自主品牌和高新技术产品出口。争创跨境电子商务服务试点城市。鼓励有条件的企业“走出去”开展国际化经营。

　　突出创新驱动。积极营造有利于万众创新的政策环境，鼓励企业加大研发投入，引导各类创新要素向企业集聚、为企业服务。全面推进“数控一代”示范工程和“泉州制造2025”及其三项行动。深化与科研院校战略合作，充分发挥中科院、工程院、华中科大等在泉创新平台的带动作用。实施传统产业智能制造、工业机器人应用开发、北斗卫星应用、数字对讲机等重大科技专项。加快国家级高新区建设。实施市级重点技改项目200项以上。深化品牌、商标、质量、标准战略，确保通过国家知识产权示范城市复核，争创全国质量强市示范市。

　　强化龙头带动。加快推进中化乙烯、泉港苯乙烯、晋江龙峰纺织等项目，培育龙头企业200家以上，支持开展合资合作、资产收购、股权置换。实施产业链建设工程，瞄准关键节点和缺失环节，实施一批上游关键材料、核心部件和下游终端产品项目。扶持中小企业走差异化、个性化、专业化发展的路子。深化“手拉手”行动，促进专业分工，做大配套产业，提高区域行业协作水平。建好国家级、省级开发区，加快泉港、泉惠石化园区整合提升，打造新的产业增长极。

　　拓展新产业新业态。培育壮大集成电路、新能源、新材料等战略性新兴产业。深化“第三产业发展年”活动，实施重点项目485个，年度计划投资725亿元。主动迎接后互联网时代的到来，推进与工业、农业以及金融等服务业的全面融合，加快并规范发展互联网经济。抓好泉州软件园、EC产业园、菜鸟智能物流、中兴海丝路电商、民生电商等项目。启动创建“中国快递示范城市”，促进物流、会展、服务外包、工业设计与实体经济有机融合。大力发展体育产业和健康服务业。深入挖掘文化、生态等旅游资源，抓好“海丝”世博城、崇武半岛国际度假区、九仙山旅游风景区、晋江五店市等项目，引导各地打造各具特色的旅游景区，打响“文都”、“海丝”、“海峡”等旅游品牌。加快发展海洋经济，做大做强临港工业、港口物流、海洋生物医药等产业，力争海洋生产总值达1800亿元。

　　加强人才支撑。充分发挥企业家才能，支持和引导民企代际传承、创业创新。深化“海纳百川”人才聚集计划，务实研究出台有利于人才“落地安心”的政策措施。完善柔性引才机制，用好博士后工作站、院士专家工作站等平台。持续实施泉籍优秀人才、留学人员回归工程。健全重点企业人才服务制度，扶持发展人力资源服务业，完善职业经理人制度。加强人才培养和技能人才队伍建设。健全以激励创新和产业化业绩为评价导向的选才用才机制。

　　（二）大力推进农业现代化，进一步激发农村发展活力

　　发展特色现代农业。稳定粮食生产，保障粮食安全。加快转变农业发展方式，强化农业科技服务，壮大绿色有机农业、特色高优农业，拓展“种养加销”全产业链经营模式。大力发展设施农业，建设一批园艺、畜禽、林业、休闲农业示范基地。发展生态循环农业，推进一批示范项目。培育新型农业经营主体和新型农民，重点扶持200家农业龙头企业。发展农村电商、农村普惠金融和农业保险，发挥供销系统和农民合作社的作用。抓好抛荒山垅田复垦和标准农田建设，加强农产品质量安全和重大动植物疫病防控。壮大远洋渔业，鼓励知名休闲食品企业从事海产品深度加工。

　　深入推进新农村建设。大力实施“千村整治、百村示范”工程，抓好农村人居环境整治和美丽乡村示范村培育，建立农村垃圾污水处理长效机制，打造一批乡村旅游景点和线路。致力增加农民经营性、财产性收入，发展壮大农村集体经济。推进山美水库生态保护试点、七库连通工程等116个水利项目，深化白濑水利枢纽工程前期工作，加强农村小型水利工程建后管护。加快农村公路提级改造。保护好乡土文化设施，留住乡愁记忆。关爱农村“三留守”群体，进一步保障农村基本公共服务。

　　更加突出精准扶贫。对接落实赣闽粤原中央苏区振兴发展规划。加强科学扶贫、产业扶贫、社会扶贫，进一步强化对口帮扶。统筹用好市级1亿元扶贫开发专项资金，对30个扶贫开发重点乡镇、300个重点村实行更具针对性的帮扶，实现年度脱贫5万人以上。完成省市造福工程危房改造建设年度任务。

　　（三）大力推进新型城镇化，进一步推动环湾向湾同城发展

　　完善立体交通网络。进一步撑大城市骨架，拓展与国内外主要城市的互联互通。开工建设吉永泉铁路、中化铁路专用线，加快湄洲湾南岸铁路支线建设。确保泉州湾跨海大桥、安溪连接线竣工通车，实现环城高速全线贯通；加快厦沙高速公路建设，力争泉厦漳联盟高速公路开工，抓好国省干线和重要交通节点项目。加快建设肖厝5-7＃、石湖5-6＃、秀涂16#等泊位。

　　合力建设环湾城市。深化“环湾规划建设年”活动，实施项目458个，年度计划投资510亿元。推进“多规合一”，抓好各项规划的衔接、管控和落实，统筹环湾区域重大产业、基础设施、民生事业等项目布局。加快新区繁荣和“三旧”改造。抓好北峰-城东通道、金屿大桥、洛江西环路等环湾路网建设，抓紧轨道交通前期工作。强化产城融合，完善中心市区“优二进三”政策，推进东海、晋江滨江、鲤城、洛江等总部区建设，培育金融区、商贸聚集区，推动房地产业健康发展。科学开发利用城市地下空间。深入实施市政基础设施“五千工程”，推进市区供水管网改造，加快晋江洛阳江两岸景观整治，实施中心市区沿江及高速公路通道两侧夜景提升工程，抓紧泉州植物园建设，启动绿道山线项目，新增城市绿地310公顷。

　　统筹推进城镇发展。加强泉州南翼和北翼新城的规划建设。抓好新型城镇化各级各类试点，推进基础设施、城市管理、环境保护一体化和基本公共服务均等化，组织实施省市试点小城镇项目600个以上。深化城乡建设“五项行动”、城市“三边三节点”整治，延伸拓展“点线面”攻坚。落实户籍制度改革，完善居住证制度，有序推进农业转移人口市民化。加快石结构房屋改造步伐，确保完成3200万平方米年度改造任务。

　　精细抓好城市管理。建立完善全国文明城市这一崇高荣誉的管理维护长效机制。加强“数字城管”建设，提高城市智能化、网格化管理水平。理顺市区两级管理职责，强化城乡综合管理考评、市容市貌观察团督查。健全三级物业协调管理体系，加强住宅专项维修资金管理，推进现有住宅加装电梯。创建节水型城市。大力发展公共交通，综合治理城市交通拥堵，加强公共停车场建设管理。启动建设中心市区公共自行车交通系统。继续抓好防洪排涝、管网清淤、内沟河截污及路面改造。加强城乡抗震设防和应急响应体系建设。保持“两违”整治的高压态势。

　　（四）大力推进改革开放，进一步加快建设“海丝”先行区

　　深化体制机制创新。要围绕建立健全实体金融的服务体系，深化金融改革。支持在泉金融机构发展，做大做强泉州银行，争取获批民营银行；大力发展产业链金融、互联网金融、小微金融、消费金融；加快中小微企业信用信息交换共享平台扩容升级和拓展应用，开展小微企业贷款保证保险；试点开展泉台跨境人民币贷款业务，探索实行外商投资企业外汇资本金意愿结汇，争取非金融机构开展跨境电子商务外汇支付业务；支持企业规范上市，力争上市企业突破100家，“新三板”挂牌企业突破10家，海峡股权泉州交易中心挂牌企业突破400家；规范发展民间金融；用好各类风险补偿资金池，强化企业资金链风险防控应急处置，严守金融安全防线。要与时俱进丰富“晋江经验”的内涵，深化民综改革。大力营造更加便利宽松的大众创业环境；深入推进三年实施计划，抓紧在基础设施、公用事业等领域推出一批重大项目，鼓励和吸引社会投资；扩大政府购买服务试点范围；深化公共资源市场化配置；加强工商登记制度改革后续监管工作，推进企业登记“三证合一”；重视发挥商会、协会的作用，规范发展社会中介组织；持续推进县域特色改革。要务实推进其他改革。推动土地承包经营权确权登记发证和依法有序流转，鼓励农民参与股份制合作；开展农村宅基地、集体建设用地使用权确权登记发证，探索建立城乡统一的建设用地市场，抓好农村产权抵押融资。有序推进不动产统一登记。抓好阶梯水价、气价改革。加强国有资产监管，提升国有资产集中度和带动能力。对接落实财税改革政策，完善地方政府举债融资和管理机制，防控政府性债务风险。

　　深入实施“海丝”战略。认真落实国家“一带一路”战略规划和福建核心区建设实施方案，启动我市“海丝”先行区建设十大行动计划、十项重点工程。大力推进泉州港复兴，加快规划建设“海丝”国际枢纽港，支持泉州港、泉州晋江国际机场开拓国际航线，进一步促进口岸通关便利化。强化双向投资贸易，发挥“海丝”国际品牌博览会、泉州品牌“海丝行”等平台作用，推动与“海丝”沿线国家和地区共同设立经贸合作区，规划建设绿色石化产业合作园区。完善国际友城网络，推动建立21世纪海上丝绸之路城市联盟和商务合作机制，拓展城市、港口、金融、文化、旅游等多领域对接。建设南洋华裔族群寻根谒祖等平台，汇聚侨心、发挥侨力，鼓励广大侨亲参与“海丝”先行区建设。推进泉港澳合作，建立“双延伸”联络协调工作机制。进一步挖掘保护“海丝”遗产，加快联合申报“世遗”进程，筹划建设“海丝”国际文化交流展示中心。复制推广国内自贸区创新成果，增创开放型经济新优势。

　　拓展泉台交流合作。加快台商投资区开发建设，大力引进台资龙头企业、先进技术和管理经验。支持台资企业转型升级，促进泉台双向投资。进一步发展对台海上、空中直航，扩大对台贸易。建好台湾农民创业园，办好涉台经贸活动。加强对台民间交流，深化科技、人才、教育、卫生、文化等领域合作。

　　（五）大力推进文化繁荣，进一步打造共同精神家园

　　保护延续城市文脉。办好第十四届亚洲艺术节，建设亚洲艺术公园。实施西街古城保护改造、市公共文化中心等一批文化基础设施项目。抓好泉州及崇武、丰州、永宁古城和蔡氏古民居保护整治，加强对历史文化名城、名街、名镇、名村、名人故居保护，开展传统村落、传统民居普查测绘。组织修编《泉州市志》。

　　做足弘扬闽南文化文章。出台闽南文化生态保护区总体规划我市的实施方案，提升“十百千”基础工程。支持申报国家级、世界级“非遗”项目，建设闽南文化生态园。完成17个传统工艺美术濒危艺种的抢救保护任务。抓好祖地文化保护建设。推进民间文化创新和民间文化活动，推动泉州文化进校园、进企业、进乡村。支持惠安申报世界石雕之都、德化申报世界陶瓷之都、永春举办世界白鹤拳大会。参与办好“东盟福建周”，加强对外文化交流合作。

　　更加注重文化惠民。践行社会主义核心价值观，深化群众性精神文明创建活动，提高市民文明素质。深入创建全国公共文化服务体系示范区，改造提升乡镇（街道）文化站，推广建设数字农家书屋，实现行政村文体活动室全覆盖。鼓励文艺创作，推出一批人民群众喜闻乐见的文艺精品。扶持国家广告产业园、源和1916、惠安雕艺文创园、印象5号博物馆等文化产业基地发展。

　　（六）大力推进绿色发展，进一步创建生态文明示范区

　　强化污染综合整治。深化工业污染治理，整合提升电镀集控区，实现所有工业园区污水集中处理和达标排放。实施更严厉的水资源保护，认真落实“河长制”，抓好重点流域、跨境流域整治以及小流域“赛水质”活动。加快污水处理厂及配套管网建设运营。有效治理生猪养殖等面源污染。加强工业源、餐饮油烟、扬尘、垃圾焚烧等大气污染整治，2005年底前注册营运的黄标车今年内要全部淘汰，完成建陶业LNG替代。开展危化品经营整治，推进石化园区应急救援中心、工业危废处置中心建设。认真落实新《环保法》，推动实施企业环境污染责任保险，依法严厉打击环境违法行为。

　　节约集约利用资源。实施重点节能项目50个以上，抓好134家重点用能企业管理，严把新上项目能耗关。抓好可再生能源建筑应用示范城市建设。推进泉港、泉惠石化园区以及德化陶瓷产业园、石狮染整工业园循环化改造，抓紧海西再生资源“城市矿产”示范基地建设。启动中心市区垃圾处理产业园项目。积极发展农村沼气工程。推进节约集约用地，严守耕地保护红线。

　　加强生态系统建设。编制生态文明建设示范区规划，巩固提升国家级生态市创建成果。完善“两江”流域生态保护补偿机制，落实生态红线管控措施。积极创建国家级森林城市，抓好造林绿化、水土保持和“青山挂白”治理，加强山体、水域、海岸带生态修复，突出重点生态功能区建设和生物多样性保护。推进森林生态景观提升和森林公园建设。抓好崇武国家级海洋公园项目。

　　（七）大力推进社会和谐，进一步构建民生保障安全网

　　切实保障基本民生。坚持保基本、兜底线、补短板，确保新增财力更多用于民生。抓好重度失能老人政府购买服务、妇婴健康工程、贫困残疾人康复救助、“儿童之家”建设、低保户安居工程、中小学幼儿园技防系统建设等39项为民办实事项目，让发展成果更多更公平地惠及全市人民。

　　提高社会保障能力。落实高校毕业生就业创业扶持政策，建设大学生创业示范基地；抓好失地失海农民、困难群众、退役士兵等就业指导帮扶，组织开展新生代农民工职业技能培训。实施“全民参保登记计划”，推进城镇职工“五险”、城乡居民基本养老保险、城镇居民医保和新农合提标扩面，稳妥实施机关事业单位养老保险并轨改革。推行居家养老、社区养老、机构养老结合，加快市社会福利中心建设，培育多功能护理型养老机构和高素质护工队伍。开展“救急难”工作，发展慈善公益和残疾人事业。深入实施妇女儿童发展纲要。完善住房公积金制度，抓好保障性安居工程建设。

　　促进教育全面发展。深化教育领域综合改革，全面推进素质教育。实施第二轮学前教育三年行动。推进城镇中小学扩容，加快城东、东海、江南学园后续建设，完善招生机制，保障适龄儿童就近入学，全面完成义务教育标准化建设，力争所有县（市、区）通过国家义务教育基本均衡认定。支持高等教育内涵发展。深化产教融合、校企合作，启动示范性职业教育集团建设。加强青少年德育和体质健康工作。重视师德教育。规范发展民办教育，实施特教三年提升计划，抓好老年教育、继续教育。加强校园安全。抓好关心下一代工作。深化全民健身运动，办好第十届市运会，做好第一届全国青年运动会泉州赛区筹备工作。

　　提升卫生健康事业。继续深化医改工作，深入实施公立医院和基层医疗机构综合改革，推进医疗、医保、医药联动，进一步解决群众看病难、看病贵问题。更加重视和加大对卫生事业的投入，大力加强卫生基层基础，尽力扩充急需的医疗资源，新增医疗机构床位2000张以上，加快培养各层级各相关专业急需的卫生人才，提高医疗技术和服务水平。构建和谐医患关系。鼓励社会资本办医。加强传染病和慢性病防控。落实人口计生政策，严格计生考核，加强计生特殊家庭保障，发挥计生协会作用，促进人口长期均衡发展。

　　创新提升社会治理和服务能力。开展重大决策社会稳定风险评估，运用好“大调解”机制和信访“路线图”。深化社会治安综合治理，完善立体防控体系，依法打击违法犯罪活动，创新社区矫正服务管理。深入实施安全生产标准化提升工程，推进安全生产隐患排查治理体系建设，持续深化道路交通和消防安全综合整治，争创安全发展示范城市。切实强化食品药品安全监管，抓好食品放心工程和药品安全专项整治，开展农产品质量安全县和食品安全城市创建活动。提高防灾减灾和应急处置能力。加快社会信用体系建设和信息应用。依法有序开展村级组织换届。支持工会、共青团、妇联等群团组织履行职能。认真贯彻中央民族工作会议精神和党的宗教工作基本方针，扎实做好民族宗教工作。统筹抓好援藏、援疆、援宁及山海协作等工作。持续推进军民融合深度发展，进一步规范国防动员与后备力量建设运行机制，争创全国双拥模范城“八连冠”。

　　三、全面提升法治政府和服务型政府建设水平

　　各位代表！新一轮跨越发展时不我待，“法治泉州”建设任重道远，我们必须主动适应新常态，积极应对新挑战，全面提升政府驾驭复杂局面的能力、组织推动发展的能力。

　　我们要抢抓机遇、奋发有为。十分珍惜和用好用足“海丝”、“文都”、金改区、综改区等“国字号”招牌，让优势有效叠加、机遇尽快转化、红利充分释放。注意顶层设计和基层探索良性互动，更好地把中央要求、群众期盼、实际需要和试点先行结合起来，努力形成经验、形成更大的效应。树立强烈的目标意识、责任意识和担当意识，高起点、高标准、高水平编制“十三五”规划，保持高于全省平均水平的发展速度。以踏石留印、抓铁有痕的干劲，推动跨越发展行动方案的落实。

　　我们要厉行法治、依法行政。强化法治思维，在党的领导下，依宪依法行政。认真执行市人大及其常委会决定决议，及时向市人大及其常委会报告工作，主动接受市人大及其常委会法律监督和工作监督，认真办理人大议案和代表建议。自觉接受市政协的民主监督，健全重大事项事前协商制度，认真办理政协提案。广泛听取民主党派、工商联、无党派人士意见建议，认真接受公众监督和舆论监督。加强协商民主建设。加快政府职能转变，深化简政放权，建立“三张清单”制度。健全依法决策机制。按市委和市人大的要求，做好取得地方立法权的准备工作。推进行政执法体制改革。加强行政复议和行政应诉工作。全面推进政务公开。完成“六五”普法目标任务，增强全民法治观念。推进四级行政服务标准化无缝对接、上下联动和乡镇全覆盖，推广应用电子证照，实现网上办理率超过80%，力促“审批再瘦身、服务再提速、中介再规范”。完善政企互动机制，建立收费目录清单制度，切实减轻企业负担，及时为企业排忧解难。

　　我们要严明纪律、清正廉洁。牢记作风建设永远在路上，认真开展“三严三实”教育，坚决反对“四风”，坚持“四下基层”。落实党政机关厉行节约反对浪费条例，推进公务用车制度改革。深化事业单位清理规范和分类改革，健全人员聘用和岗位管理制度。落实县以下机关公务员职务与职级并行制度。各级政府及其各部门的党组（党委）要切实履行党风廉政建设的主体责任，加强薄弱领域和关键环节存在问题的整改整治，更加重视制度反腐，抓好风险防控。加强行政监察和审计监督。推行干部绩效考评对账制度。在位就要有状态，为官必须敢担当。全体公务人员一定要讲政治、讲规矩，常警醒、知敬畏、听招呼，进一步提振干事创业激情，进一步坚守法纪底线，真正做到既干净又干事，既廉洁又勤政。

　　各位代表！新的时代赋予我们新的使命，未来召唤着我们开拓奋进。让我们紧密团结在以习近平同志为总书记的党中央周围，在中共泉州市委的领导下，紧紧依靠全市人民，解放思想，凝心聚力，只争朝夕，锐意进取，为让泉州人民过上更加安宁富足的生活而努力奋斗！
